# Serum Neuropeptide FFR2 is increased in pregnant women with pre-eclampsia and associated with pregnancy outcomes

**DOI:** 10.12669/pjms.41.2.10591

**Published:** 2025-02

**Authors:** Ummu Tas, Sedat Tas, Tuncay Kume, Ozgur Yilmaz

**Affiliations:** 1 Ummu Tas Associate Professor, Department of Cardiology, Izmir Demokrasi University, Goztepe, Izmir, Turkey; 2 Sedat Tas Associate Professor, Department of Cardiology, Manisa Celal Bayar University, Yunusemre, Manisa, Turkey; 3 Tuncay Kume Associate Professor, Department of Biochemistry, Dokuz Eylul University, Balcova, Izmir, Turkey; 4 Ozgur Yilmaz Department of Jinecology, Manisa City Hospital, Sehzadeler, Manisa, Turkey

**Keywords:** Neonatal outcome, Neuropeptide FF (NPFF), Neuropeptide FFR2, Pre-eclampsia, Pregnancy, Syncytin

## Abstract

**Objectives:**

NPFFR2 is a biomarker produced by the placenta during pregnancy and is thought to be associated with in various physiological processes, including pain modulation, opioid receptor regulation, and cardiovascular function. Pre-eclampsia (PE) is a major public health concern due to its links with cardiovascular disease (CVD), stroke and neonatal morbidity and mortality. Consequently, timely diagnosis and efficient management of PE are essential for both maternal and neonatal health. This study aimed to conduct a comparative analysis of neuropeptide FFR2 (NPFFR2), echocardiographic evaluation results, and pregnancy outcomes in pregnant women with and without PE.

**Methods:**

This is a prospective case-control study. It included 94 pregnant participants who applied to Manisa City Hospital between October 2021 to January 2023 and were grouped into women with PE (n = 47) and those without PE (n = 47). Biochemical and NPFFR2 analyses were performed using the blood samples collected from all participants, along with echocardiography and 24 hours. Ambulatory blood pressure monitoring (ABPM). A p-value <0.05 was considered statistically significant

**Results:**

The study group comprised 94 pregnant women with a mean age of 29.2 years and mean gestational age of 27.6 weeks. The preeclampsia group had a significantly higher NPFFR2 levels, lower gestational age at birth and higher all 24-hours ABPM findings. The left atrial-to-aortic ratio and right ventricle myocardial performance index were significantly higher and EA ratio was significantly lower in the preeclampsia group than in the control group. NPFFR2, gestational age at birth, LDL cholesterol, and body mass index were found to be independently associated with neonatal intensive care unit admission.

**Conclusions:**

The women with PE presented with increased serum NPFFR2 levels and the prognosis of pregnancy was associated with NPFFR2 levels.

## INTRODUCTION

Gestational hypertension is a condition that develops after the 20th week of pregnancy and resolves within 42 days of delivery. It occurs in 6% of pregnant women and progresses to Pre-eclampsia (PE) in the range of 15%-45%.^1^ International guidelines are consistent in defining hypertension in pregnancy as having a blood pressure (BP) of ≥140/90 mm Hg, in contrast to the American Heart Association, which defines it as a BP of ≥130/80 mm Hg.^2^-^4^ Preeclampsia that is a hypertensive condition as well as albumin protein in the urine in pregnancy is a serious public health concern, as it is a major risk contributor to CVD, stroke and peripheral vascular disease.^5^ Nearly half of the maternal deaths are attributed to the abovementioned complications related to HT, a preventable and treatable disease.^6^

Two primary receptors for NPFF have been identified, NPFFR1 and NPFFR2. The placental overexpression of NPFF via NPFFR2 is associated to HT, whereas its lack of secretion in the brain is associated with HT. Kalliomäki et al. have reported the disappearance of cells expressing NPFF mRNA from the supraoptic and paraventricular hypothalamic nuclei following salt loading.^7^ The decreased release of neuropeptides in the neurons may be associated with HT. Goncharuk et al.’s studies have indicated that the patients with HT showed a decreased NPFF expression.^8^,^9^ Aside from the brainstem, NPFF and its receptors were also highly expressed by the cytotrophoblast cells in the placenta. Zhu has observed that NPFFR2 was constantly expressed during gestation and was higher in the placenta samples acquired from women with PE in comparison with their brainstems.^10^

In accordance with Zhu et al.’s research, Jiang et al. suggested that the NPFFR2 protein in the placenta increases and is then released into the maternal peripheral blood through the maternal-fetal circulation and that increased NPFFR2 is associated with PE.^10^,^11^ Lin et al. indicated that NPFFR2 tends to exhibit a positive effect on the hypothalamic-pituitary-adrenal (HPA) axis through its impact on hypothalamic CRF.^12^ As NPFFR2 plays an important role in both HT and pregnancy, in the present study, we conducted a comparative analysis of NPFFR2, echocardiographic evaluation results, and pregnancy outcomes between pregnant women with and without PE.

## METHODS

This prospective case-control study included 94 pregnant participants who applied to Manisa City Hospital between October 2021 and January 2023. Participants were divided into two groups: women with preeclampsia (PE, n=47) and without PE (n=47), matched for age and gestational age. In determining the effect size, calculations were performed using a difference smaller than the medium effect size defined by Cohen, as the effect’s power could not be precisely estimated. Based on these assumptions, with a Type-I error rate of α=0.05 and a statistical power of 0.85, the minimum required sample size was calculated to be 82 participants, with 41 participants in each group, for an effect size of d=0.60. PE was defined as hypertension (systolic BP ≥140 mmHg and/or diastolic BP ≥90 mmHg) and proteinuria (≥300 mg/24-hour urine) after the 20th week of gestation, following current guidelines. Decisions regarding MgSO_4_, antihypertensive therapy, and pregnancy termination were based on PE severity and gestational age. Ethical approval was obtained (No: 2021/09-21), and all participants provided informed consent. Exclusion criteria included comorbidities and congenital anomalies in infants.

### Sample collection and biochemical analysis:

Morning venous blood samples were collected after overnight fasting, centrifuged, and stored at −80°C. Serum NPFFR2 levels were measured using a commercial ELISA kit (sensitivity: 15 pg/mL, range: 2.5–4000 pg/mL).

### Ambulatory BP Monitoring:

BP measurements were obtained using validated devices, with hypertension defined per guidelines. Participants were categorized as “dippers” (≥10% nocturnal BP reduction) or “non-dippers” (<10% reduction) based on 24-hour ambulatory BP monitoring. ***Echocardiographic evaluation:*** Transthoracic echocardiography was performed to assess cardiac parameters, including LV ejection fraction, LA volume index, E/A and E/Em ratios, and RV myocardial performance index.

### Statistical analysis:

Data were analyzed using SPSS v24.0. Continuous variables were tested for normality (Kolmogorov-Smirnov/Shapiro-Wilk) and compared using t-tests or Mann-Whitney U tests. Categorical data were analyzed using chi-square or exact tests. Cox regression identified risk factors for morbidity/mortality, and ROC analysis evaluated biomarker utility, reporting AUC, sensitivity, specificity, and accuracy. A p-value <0.05 was considered statistically significant.

## RESULTS

### Baseline characteristics and BP profile:

The study group comprised 94 pregnant women with a mean age of 29.2 (22-43) years and mean gestational age of 27.6 (20-37) weeks. According to the results of the 24-h ABPM, 26 pregnant women (55.4%) were dippers and 21 (44.6%) were non-dippers. There were 10 (10.6%) neonatal intensive care unit (NICU) admissions and 4 (4.3%) neonatal deaths among the participants. The HT group had significantly lower gestational age at birth (p < .001) as well as had infants with lower birth weight (p < .001). Table-I contains an overview of the demographic and clinical details of the patients.

**Table-I:** The Comparison of baseline characteristics of the participants.

	HT	χ̅±SS	X̃ [min-max]	Test statistics; p-value
Age (year)	No	28.7±5.27	29 [22-43]	t = 1.030 p = 0.30
	Yes	29.7±4.93	29 [22-43]	
Gestational age at blood draw (week)	No	28.3±4.25	28 [20-37]	t = 1.56 p = 0.12
	Yes	27.06±3.36	26 [20-35]	
Parity	No	0.72 ± 0.61	1 (0-2)	p = 0.50
	Yes	0.63 ± 0.60	1 (0-2)	
Smoking (n, %)	No	2 (4.3)		p = 0.24
	Yes	5 (10.6)		
BMI (kg/m^2^)	No	25.9±5.1	26.5 [19.5-36.8]	t = 0.516 p = 0.60
	Yes	27.5±4.3	27.4 [21.51-39.9]	
Gestationalageatbirth (week)	No	38.28±0.8	38 [37-40]	t = 18.220 p < .001
	Yes	34.19±1.31	34 [31-37]	
Birthweight (gr)	No	3172.34±127.79	3190 [2950-3450]	U = 0.001 p < .001
	Yes	2619.57±208.35	2680 [2125-2920]	

U: Mann Whitney U test statistics. t: Student’s t test statistics. p<0.05 Significance level, BMI: Body mass index, bpm: beat per minute, HR: Heart rate, OSBP: Office systolic blood pressure, ODBP: Office diastolic blood pressure.

### Comparison of independent variables:

The preeclamptic group had significantly higher NPFFR2 levels (p < .001) and higher 24-hours daytime and nighttime SBP (p < .001, p < .001) and DBP (p < .001, p < .001). Table-II shows the comparison of the NPFFR2 levels findings in the study groups. Based on the findings of the echocardiographic evaluation, LA:Ao ratio (p = .009) and RV MPI (p = 0.03) were found to be significantly higher and EA ratio (p = 0.02) was found to be significantly lower in the preeclamptic group compared with the control group. Table-III shows the comparison of the echocardiographic findings between the study groups.

**Table-II:** The Comparison of NPFFR2 levels of the participants.

	HT	χ̅±SS	X̃ [min-max]	Test statistics; p-value
NPFFR2 (pg/mL)	No	228.4±206.1	183 [1-825]	t = 3.978 p < .001
	Yes	513.9±446.8	324 [1-1922]	

U: Mann Whitney U test statistics. t: Student’s t test statistics. p<0.05 Significance level.

**Table-III:** The comparison of echocardiographic evaluation of the participants.

	HT	χ̅±SS	X̃ [min-max]	Test statistics; p-value
LVEF (%)	No	62.17±2.39	62 [58-69]	t=0.001 p=0.99
	Yes	62.17±2.44	62 [56-66]	
AORT (cm)	No	2.97±0.35	2.91 [2.19-3.73]	t = -1.414 p = 0.17
	Yes	3.01±0.3	3.04 [2.33-3.53]	
LA (cm)	No	4.12±1.05	3.55 [2.73-4.49]	t = 1.868 p=0.07
	Yes	4.4±0.97	4.0 [3.0-5.3]	
LA : Ao	No	1.19±0.18	1.16 [0.82-1.62]	t = 2.678 p=.009
	Yes	1.36±0.23	1.38 [0.82-1.94]	
LAVI (ml/m^2^)	No	32.68±12.53	34.13 [7.97-59.83]	t = 0.723 p=0.47
	Yes	34.71±14.56	33.15 [11.01-83.27]	
RV (cm)	No	3.4±0.5	3.49 [2.29-4.61]	t=0.373 p=0.71
	Yes	3.35±0.64	3.21 [2.3-5.23]	
E/A	No	1.04±0.3	0.94 [0.46-1.67]	U=791 p=0.02
	Yes	0.91±0.29	0.84 [0.53-1.84]	
E/Em	No	12.19±4.33	11.33 [5.77-25.46]	U=976 p=0.33
	Yes	14.28±7.61	12.16 [6.59-41.12]	
TAPSE	No	2.16±0.28	2.14 [1.72-2.87]	U=839 p=0.045
	Yes	2.3±0.34	2.33 [1.75-3.06]	
RV MPI	No	0.77±0.35	0.67 [0.38-1.62]	U=815 p=0.03
	Yes	0.88±0.29	0.86 [0.34-1.8]	

U: Mann Whitney U test statistics. t: Student’s t test statistics. p<0.05 Significance level. Ao: Aort, RV: Right ventricle, LA: Left atrium, LA:Ao: The ratio of left atrium to aort, LAVI: Left atrial volume index, LVEF: Left ventricular ejection fraction, PAT: Pulmonary acceleration time, E: Early peak of mitral inflow velocity, A: Late peak of mitral inflow velocity, Em: Early diastolic mitral annular velocity, E/A ratio: Ratio of early (E) to late (A) peak of mitral inflow velocity, E/Em: Ratio of early (E) peak of mitral inflow velocity to early (Em) diastolic mitral annular velocity, RVET: Right ventricle ejection time, RV MPI: Right ventricle myocardial performance index, TAPSE: Tricuspid annular plane systolic excursion.

We found no significant differences between the groups in terms of laboratory results except for ferritin (p < 0.001), aspartate (p < 0.001) and alanine aminotransferase (p<.001), calcium (p = 0.04), and low density lipoprotein (LDL-C) (p = 0.01). In Table-IV, the comparison of the laboratory findings between the study groups. The non-dipper group exhibited significantly higher NPFFR2 levels (p = 0.03), as well as higher nighttime SBP (p < .001), DBP (p = .006) and lower gestational age at birth (p = .001), compared with the dipper group. Moreover, the non-dipper group was older and had a higher BMI, EEm ratio, and LA volume index, but no significant difference was observed.

**Table-IV:** The comparison of laboratory findings of the participants.

	HT	*χ̅*±SS	X̃ [min-max]	Test statistics; p-value
Cre (mg/dl)	No	0.83±0.19	0.8 [0.52-1.22]	U=824 p=0.03
	Yes	0.92±0.2	0.92 [0.55-1.32]	
AST (U/L)	No	18.4±14.6	13 [2-64]	t = 3.714 p < .001
	Yes	43.0.±36.6	21 [7-162]	
ALT (U/L)	No	15.9±10.8	12 [2-57]	t = 4.804 p < .001
	Yes	31.9±20.1	27 [8-108]	
T- Chol (mg/dl)	No	195.06±45.23	202 [84-274]	t=0.967 p=0.34
	Yes	186.3±42.66	189 [84-275]	
HDL (mg/dl)	No	42.91±14.78	42 [10-91]	t=0.708 p=0.48
	Yes	40.98±11.53	40 [15-71]	
TG (mg/dl)	No	145.96±68.56	143 [58-474]	t = 0.032 p = 0.97
	Yes	146.47±84.18	140 [48-538]	
LDL (mg/dl)	No	125.32±39.51	129.0 [28-214]	t=2.511 p=0.01
	Yes	145.0±36.5	146.4 [57-204]	
Ca (mmol/l)	No	9.10±0.49	9.1 [7.9-10]	t= -2.029 p=0.04
	Yes	8.86±0.63	9.1 [6.6-9.9]	
	Yes	41.29±6.43	42.5 [25.4-52.8]	
Ferritin (ug/L)	No	197.72±119.25	185 [6.23-677]	U=573 p<.001
	Yes	305.16±150.27	280 [100-770]	

U: Mann Whitney U test statistics. t: Student’s t test statistics. p<0.05 Significance level, Glu: Glukose, Cre: Creatinin, GFR: Glomerular filtration rate, Alb: Albumin, AST: Aspartate aminotransferase, ALT: Alanin aminotransferase, Ca: Calcium, Mg: Magnesium, T-Chol: Total cholesterol, LDL: Low density lipoprotein, HDL: High density lipoprotein, TG:Trigliserid, WBC: White blood cell, RBC:Red blood cell, HB: Hemoglobin, HCT: Hematocrit.

### Cox analysis and ROC:

**Fig.1:**
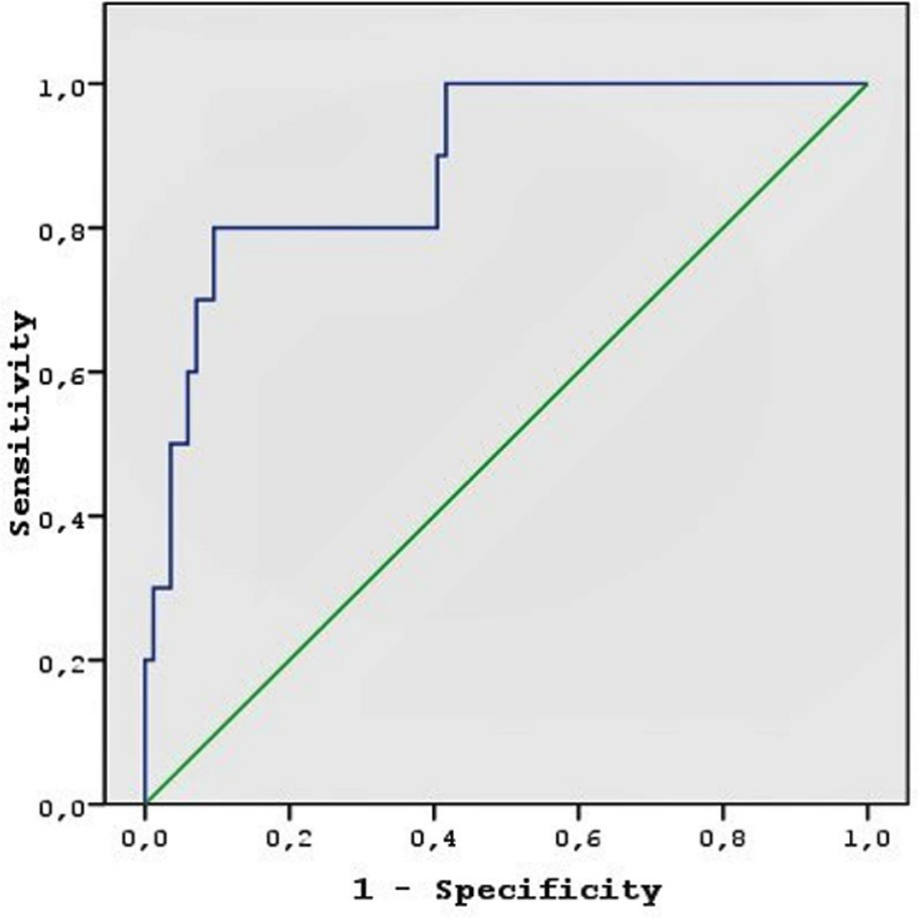
The receiver operating characteristic (ROC) curve to determine a cutoff value for identifying the relationship between Neuropeptide FFR2 levels and neonatal intensive care unit admission; the optimal cut-off value of 673.5 had sensitivity of 80% and specificity of 91%, area under receiver operating characteristic curve: 0.887.

The ROC curve analysis was performed to determine the NPFFR2 cutoff value for the prediction of NICU admission (Fig.1). The cutoff value for NPFFR2 was determined as 673.5 mmHg and the area under the curve was 0.887. At this cutoff value, the sensitivity for predicting NICU admissions was 80.0% and specificity 91.0%. We also performed a Cox analysis using NPFFR2, BMI, age, gestation age at birth, daytime and nighttime SBP and DBP, and LDL-C to predict NICU admission. NPFFR2, gestational age at birth, BMI, and LDL-C were independently associated with NICU admission. (Table-V).

**Table-V:** Cox analyses between NICU admission and independent variables.

Independent variables	OR (95%CI)	P
NPFFR2	1.011 (1.001-1.009)	0.03
BMI	1.757 (1.010-3.054)	0.04
Age	0.872 (0.687-1.107)	0.26
Gestational age at birth	0.147 (0.035-0.622)	0.009
LDL	1.106 (1.015-1.205)	0.02
Nighttime SBP	0.812 (0.653-1.009)	0.06
Nighttime DBP	2.055 (0.892-4.731)	0.09
Daytime SBP	1.476 (0.895-2.434)	0.13
Daytime DBP	0.490 (0.215-1.118)	0.09

BMI: Body mass index, DBP: Diastolic blood pressure, LDL: Low density lipoprotein, SBP: Systolic blood pressure.

## DISCUSSION

The primary findings of our study were as follows: (i) the serum NPFFR2 levels were increased in patients with PE compared with those without PE (ii) the serum NPFFR2 levels were increased in non-dipper pregnant women with PE compared with the dipper pregnant women with PE. (iii) Abnormal LV and RV diastolic function and larger LA: Ao were common in patients with preeclamptic group (iv) the cut off value of a NPFFR2 level of >673.5 was significantly associated with NICU admission. (v) NPFFR2, LDL-C, gestational age at birth, and BMI were found to be independently associated with NICU admission but not neonatal mortality.

Recent studies have explored biomarkers like NPFFR2, which is expressed in the placenta and plays a role in cytotrophoblast differentiation.^13^-^15^ Our study found elevated NPFFR2 levels in preeclamptic women, especially in those with non-dipping status, and these higher levels were associated with worse pregnancy outcomes.

Numerous studies have exhibited the relationship between low syncytin 1 and syncytin 2 levels and pre-eclampsia and its severity.^16^,^17^ Knerr et al. suggested that reduced syncytin expression may contribute to placental dysfunction and altered placentogenesis in PE.^18^ Since NPFFR2 induces syncytin-1 and 2, a decrease in syncytin-1 and 2 may lead to a compensatory increase in the serum NPFFR2 levels in preeclamptic patients. Thus, it can be suggested that increased NPFFR2 expression caused by deficient levels of syncytin could be a contributory factor for the diminished arrangement of syncytiotrophoblasts and impaired arrangement of whole placental villi. PE is characterized by hypoperfusion and villous hypoxia that are consequences of altered trophoblast differentiation and vascular dysfunction.

Other possible mechanisms associated with PE are inflammation and oxidative stress. Inflammatory events comprising of various cell types and molecules have been associated with hypertension. Sun et al. suggested that NPFF has a potential role in the anti-inflammatory field both in vitro and in vivo.^19^ In line with Sun et al.’s research, Wagas et al. showed that NPFF treatment increased interleukin-10 transcription and abolished interleukin-6 and tumor necrosis factor-alfa transcription and NPFFR2 was upregulated by interleukin-4, an inflammatory cytokine.^19^,^20^ We believe that increased NPFF as well as NPFFR2 levels may be a compensatory response to inflammation as a component of hypertension etiology.

The pathophysiology of NPFFR2 in PE might be affected through multiple channels. Taken together that NPFFR2 signaling involve the activation of the HPA axis, which is centrally implicated in the pathophysiology of PE, it is conceivable that NPFFR2 can cause structural and functional alterations, mainly manifesting as vascular remodeling via HPA axis pathway, such that as NPFFR2 increases, HPA axis is overactivited, which seems to cause incompletely vascular remodeling. In our study, we found that the participants with PE had significantly higher serum NPFFR2 levels. To date, no study has investigated the relationship between serum NPFFR2 levels, PE and pregnancy outcomes.

Pregnancy-induced hypertension (PIH) causes dramatic changes in a woman’s cardiovascular system, which can be accessed via echocardiography. Ye et al. showed that women with PIH had significantly larger LA and more decreased LA function and that LA enlargement was associated with worse outcomes.^21^ Consistent with the abovementioned studies, we also found that LA was larger in women with PE. Furthermore, in contrast to previous studies, we found that LA:Ao was significantly higher in women with PE and associated with circulating NPFFR2 levels. Considering the LA and Ao diameter separately, no significant differences were observed between the groups, while LA:Ao was significantly higher in women with PE and associated with high circulating NPFFR2 levels.

In our study, aside from NPFFR2 level and gestational age at birth, we also observed that BMI and LDL-C were linearly associated with NICU admission. In a recent review, Tesfa et al. suggested that TG, LDL-C, and Total-C were associated with the risk of PE.^22^ In another review, Mészáros et al. suggested that utilizing pravastatin as a preventative treatment may lower the risk of pre-eclampsia as well as decrease the odds of preterm birth and NICU admission in newborns.^23^

NPFFR2 is a biomarker that has not yet been studied in pregnant women, and our study represents the first investigation of this topic in the literature. The results of our study suggest that NPFFR2 may be associated with preeclampsia. This biomarker, which we found to be significantly elevated in the serum of preeclamptic patients and associated with pregnancy prognosis, could serve as a practical agent for preeclampsia screening. However, close monitoring and obtaining serum NPFFR2 levels at different stages of pregnancy are needed to enable more robust studies in the future

### Limitations:

It includes a small sample size. Second, NPFFR2 is known to activate syncytin-1 and syncytin-2 and anti-inflammatory mediators. We hypothesized that NPFFR2 may be increased in PE secondary to reduced syncytin-1 and syncytin-2 and increased inflammatory mediators. However, it has not been determined whether the effects of increased NPFFR2 on PE were mediated via syncytin-1 and syncytin-2 and inflammation since these cytokines were not assessed in the present study. Third, the generalizability of this study was limited due to the fact that we included uncomplicated essential hypertensive individuals and did not include patients with additional co-morbidities, such as diabetes, heart failure, cerebrovascular disease, or chronic kidney disease. Fourth, it would be inappropriate to interpret the statistical insignificance of certain potential risk factors for neonatal mortality as evidence of their absence as risk factors in individual patients.

## CONCLUSIONS

NPFFR2 is a simple and cost-effective biomarker which could be used in PE, such a terrible health problem due to poor outcomes. Furthermore, we believe that this research may provide sufficient evidence for NPFFR2 as having a potential role in the development of PE.

### Author’s Contributions:

**ST:** Study conception and design, data collection and writing manuscript. **UT:** Analysis and interpretation of results, writing manuscript and draft manuscript preparation. **OY:** Data collection and writing manuscript. **TK:** Analysis and interpretation of results and writing manuscript. All authors reviewed the results and approved the final version of the manuscript. They are also accountable for the integrity of the study.
